# The High Levels of Soluble Receptors for Tumor Necrosis Factor and Heart Injury in Children with the Pediatric Inflammatory Multisystem Syndrome Associated with Coronavirus Infection: Is This Just a Coincidence? A Proof-of-Concept Study

**DOI:** 10.3390/ijms26030924

**Published:** 2025-01-22

**Authors:** Maciej Marczak, Alicja Krejner-Bienias, Agnieszka Jasińska, Marek Kulus, Paweł Miklis, Katarzyna Grzela, Tomasz Grzela

**Affiliations:** 1 Department of Pediatric Pulmonology and Allergy, Medical University of Warsaw, 02-091 Warsaw, Poland; 2 Department of Histology and Embryology, Medical University of Warsaw, 02-004 Warsaw, Poland

**Keywords:** COVID-19, coronavirus disease, heart injury, NT-proBNP, PIMS, pediatric inflammatory multisystem syndrome, TNF, tumor necrosis factor, TNF-R, tumor necrosis factor-receptor

## Abstract

(1) Pediatric inflammatory multisystem syndrome (PIMS) is a relatively rare complication of coronavirus disease (COVID-19). So far, it is unclear why COVID-19 in children has usually mild or asymptomatic courses, whereas PIMS, which develops several weeks after COVID-19, is a serious life-threatening condition. (2) In this proof-of-concept study, using the ELISA method, we compared selected clinical and immunological parameters in small groups of children with PIMS and COVID-19. Children with various inflammatory diseases were included as a control. (3) Patients with PIMS revealed significantly higher levels of pro-inflammatory molecules (C-reactive protein and IL-6) and markers of heart injury (troponin I and N-terminal prohormone of brain natriuretic peptide) as compared to other groups. Moreover, these markers correlated with increased levels of soluble receptors for tumor necrosis factor (sTNF-R1 and sTNF-R2). (4) Our observation may be a step forward to better understand the phenomenon of mild COVID-19 in children and its severe complications in PIMS. It is hypothesized that the delayed inflammation results in excessive cardiomyocyte damage and the release of sTNF-R1 and -R2. Therefore, possibly the involvement of the TNF pathway in PIMS could be explored as a potential therapeutic target. However, further studies are required to validate this approach.

## 1. Introduction

Pediatric inflammatory multisystem syndrome (PIMS) associated with coronavirus infection is a systemic inflammatory disease, also known as multisystem inflammatory syndrome in children (MIS-C), which was described for the first time in May 2020 [1]. It is estimated that PIMS/MIS-C develops in 1 in 1000 children with coronavirus [2], and its development results from dysregulation of the immune system that occurs several—usually three to four and up to six—weeks after mild or asymptomatic coronavirus infection [3]. The diagnosis is based on the history of coronavirus disease (COVID-19) or virus exposure (confirmed with antibody tests), specific clinical symptoms (high fever, abdominal discomfort/pain, skin rash, conjunctivitis, fatigue), and elevated levels of inflammatory markers, similar to those reported in a “cytokine storm” in an adult’s COVID-19, predominantly interleukin-6 (IL-6) [4].

IL-6 belongs to the most important regulatory factors in the immune system. It is produced mainly by monocytes and macrophages and has pleiotropic effects on different cell types. IL-6 initiates the immune response, stimulates B cells to produce immunoglobulins, and induces the differentiation of Th17 lymphocytes [5]. The results of numerous studies showed markedly elevated levels of IL-6, which is useful as an inflammatory marker in the diagnosis and assessment of disease severity [6,7,8]. Therefore, since IL-6 has previously been implicated in severe COVID-19, it may possibly also be involved in the inappropriate hyperactivation of immunity in PIMS/MIS-C. Of note, the biological agents that block the IL-6 pathway are currently considered as one of therapeutic options in PIMS/MIS-C [4].

There are several knowledge gaps existing in PIMS pathophysiology. First of all, it is unclear why the initial course of COVID-19 in children is rather mild, whereas its later phase, PIMS, is associated with serious life-threatening complications. The next unresolved question is the pathomechanism of the most serious PIMS complications predominantly affecting the cardiovascular system. They include acute myocarditis with left ventricular dysfunction, dilatation or aneurysm formation in coronary arteries, pericardial effusion, hypotension, and shock [9].

The aforementioned complications in PIMS may suggest the involvement of several molecules, which play a role in cardiovascular homeostasis but also reveal some immunoregulatory properties. Among possible candidates displaying such two-directional action is adenosine deaminase (ADA) [10]. ADA is an enzyme that irreversibly deaminates adenosine to inosine. Since adenosine reveals some anti-inflammatory activity, the maintenance of its concentrations and activity seems to be essential for the protection against endothelial dysfunction and vascular inflammation. Indeed, the upregulated ADA activity and decreased levels of adenosine were reported in several cardiovascular pathologies, including atherosclerosis, acute myocardial infarction, myocardial ischemia–reperfusion injury, hypertension, and thrombosis [11]. Moreover, due to the immunosuppressive action of adenosine against T cells, ADA is thought to play an important role in cell-mediated immunity by promoting the proliferation, differentiation, and maturation of lymphocytes [12]. An increase in ADA concentration and activity was observed in several inflammatory diseases, e.g., rheumatoid arthritis [10,13]. It is plausible that ADA might also be involved in COVID-19 and PIMS immunology.

Other molecules with such dual properties could be the soluble forms of receptors for tumor necrosis factor (sTNF-R1 and sTNF-R2). They both are the cleaved-off portions of surface TNF-Rs, which may act as decoys or neutralizing molecules against TNF, thus modulating the signaling pathways of this pro-inflammatory cytokine [14]. The high levels of soluble TNF receptors were reported in various conditions, including infections, autoimmune diseases, or malignancy [15]. Also, in adults with COVID-19, the levels of sTNF-R1 and sTNF-R2 correlated with disease severity [16], and the highest concentrations of both molecules were observed in patients admitted to the intensive care unit [17]. Recently it has been found that the serum levels of both soluble TNF receptors could also be used as a sensitive marker of heart injury, since they increase after myocardial infarction and, moreover, that increase correlates with infarct size and left ventricular end-diastolic volume [18]. Furthermore, high levels of sTNF-R1 and sTNF-R2 may be associated with mortality in patients with ST-segment elevation myocardial infarction (STEMI) [19]. One could hypothesize that soluble TNF receptors might also be engaged in the phenomenon of post-COVID-19 course toward PIMS in children, particularly contributing to the pathomechanism of serious cardiovascular complications in these patients. However, no such studies have been undertaken so far.

Therefore, the aim of our proof-of-concept study was to assess the serum levels of the well-known immunoregulatory cytokine IL-6 but also the aforementioned “bi-directional” molecules, among them ADA, as well as both soluble TNF receptors, and the aim was also to analyze their possible association with some clinical parameters in small group of children with COVID-19 and PIMS. A small heterogeneous group of patients with various non-COVID-19 inflammatory diseases served as a control.

## 2. Results

This proof-of-concept study involved group of 60 children, either with a confirmed diagnosis of COVID-19 (*n* = 28), PIMS (*n* = 14) or other non-COVID-19 inflammatory (INFLAM) diseases (*n* = 18). With non-equal variances and a very small size of patient groups, some of the tested data did not conform to a normal distribution. The short summary of demographic and clinical features of study groups is shown in Table 1.

The patients from the COVID-19 group were relatively younger (mean age 5.6 ± 6.2 years) as compared to others (mean 7.8 ± 4.8 years for PIMS and 6.1 ± 4.8 years for INFLAM group), but this difference was non-significant. More than half of them were younger than 2 years, whereas nearly one-third of patients in this group were older than 8 years. Despite high fever (mean body temperature 38.7 ± 0.9 °C) and significantly decreased tissue oxygenation (mean saturation 93.6 ± 6.5%), all patients from the COVID-19 group presented a relatively mild course of disease. Besides mild-to-moderate gastric symptoms (mainly abdominal pain) in less than one-fifth of the patients from this group, one-fourth of them revealed mild pulmonary injury, as verified in chest computed tomography. Only one patient from this group displayed features of benign cardiomyositis with moderate arrhythmia and slightly increased fatigue.

In contrast to the aforementioned, patients from the PIMS group revealed a more severe course of disease. Apart from higher mean body temperature (39.5 ± 0.9 °C), all patients presented some gastric symptoms (abdominal pain with diarrhea, nausea, and/or vomiting) of various intensity, headache, and fatigue. Nearly one-fifth of PIMS patients displayed mild pulmonary injury confirmed in chest computed tomography. In two-thirds of the patients, some skin symptoms (mainly skin rash or erythema) were observed. Notably, more than three-fourths of the patients from this group presented moderate-to-severe symptoms of heart injury with arrhythmia, hypotension, chest pain, edema, and increased fatigue.

The third group, designated as a non-specific control, intentionally was highly heterogeneous and involved patients with various diseases of inflammatory background but with excluded coronavirus etiology. The mean body temperature in the INFLAM group (38.2 ± 0.8 °C) was lower as compared to other groups, although this difference was non-significant. Nearly half of the patients from the INFLAM group suffered from moderate-to-severe infection of the respiratory tract, mainly of viral etiology, except for three cases with moderate pulmonary injury, as verified in chest computed tomography. One-fourth of the patients from the INFLAM group revealed mild gastrointestinal symptoms, two patients presented moderate urinary tract infection, another two with acute otitis media, two with moderate cardiomyositis, and one patient with mild arthritis.

**Figure 1:**
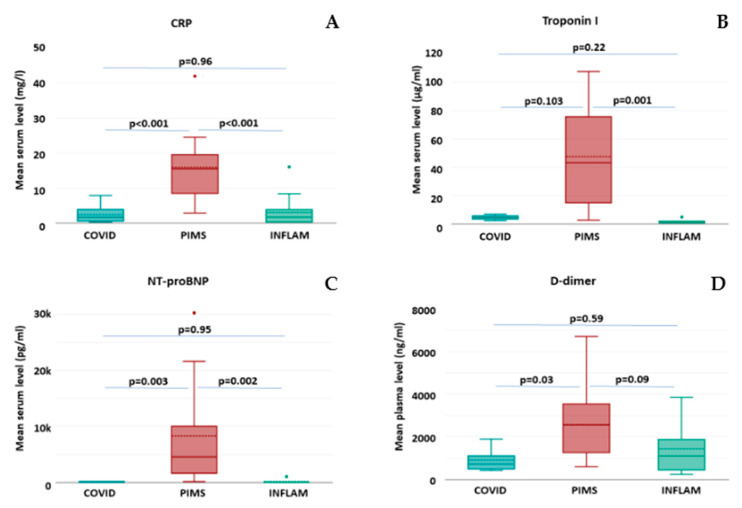
The concentrations of selected laboratory markers in study groups of COVID-19 (*n* = 28), PIMS (*n* = 14), and INFLAM (*n* = 18). The box plots represent the standard data structure; the boxes correspond to the interquartile range (with quartile Q1: lower edge; Q2 or median: solid line within the box; dashed line: mean; and Q3: upper edge,) and whiskers correspond to “non-outlier” minimum and maximum values. Each panel shows results for respective markers. (**A**) C-reactive protein; (**B**) troponin I; (**C**) N-terminal prohormone of brain natriuretic peptide; and (**D**) D-dimer. The differences between groups with *p* < 0.05 are considered statistically significant.

The comparison of selected parameters from routine blood tests did not show any significant differences in the mean numbers of total white blood cells (WBCs) and platelets (PLTs), as well as mean concentrations of hemoglobin (Hb) and creatinine (Table 2). The mean concentration of C-reactive protein (CRP) was significantly higher in the PIMS group compared to either COVID-19 or INFLAM patients (Table 2 and Figure 1A).

The assessment of laboratory markers of cardiovascular injury revealed significantly higher mean concentrations of troponin I (Figure 1B), N-terminal prohormone of brain natriuretic peptide—NT-proBNP (Figure 1C), and the product of fibrin degradation, D-dimer (Figure 1D), in the PIMS group as compared to COVID-19 or INFLAM patients. Interestingly, no significant differences in the mean levels of the abovementioned markers were observed between COVID-19 and INFLAM groups.

The analysis of selected immunological factors was shortly summarized in Table 3.

**Figure 2:**
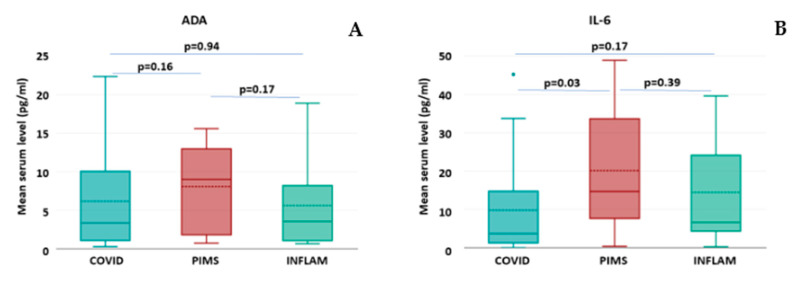
The concentrations of selected immunomodulators in study groups of COVID-19 (*n* = 28), PIMS (*n* = 14), and INFLAM (*n* = 18). The box plots represent the standard data structure; the boxes correspond to the interquartile range (with quartile Q1: lower edge; Q2 or median: solid line within the box; dashed line: mean; and Q3: upper edge,) and whiskers correspond to “non-outlier” minimum and maximum values. Each panel shows results for respective molecules, namely (**A**) adenosine deaminase; (**B**) IL-6. The differences between groups with *p* < 0.05 are considered statistically significant.

It was shown that patients from the PIMS group presented the highest mean and median values among all study groups for all tested molecules, although in the case of ADA, this difference did not reach statistical significance (Table 3, Figure 2A). The assessment of serum concentrations of IL-6 revealed that in all tested groups, the mean levels of this cytokine were above the normal limit (>1.9 pg/mL); In the PIMS group, the levels were the highest, in INFLAM, they were intermediate, whereas in COVID-19, they were the lowest. However, the difference appeared statistically significant only between the COVID-19 and PIMS groups (Table 3, Figure 2B).

**Figure 3:**
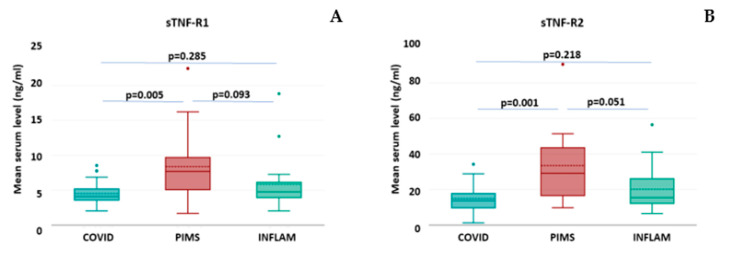
The concentrations of soluble TNF receptors in study groups of COVID-19 (*n* = 28), PIMS (*n* = 14), and INFLAM (*n* = 18). The box plots represent the standard data structure; the boxes correspond to the interquartile range (with quartile Q1: lower edge; Q2 or median: solid line within the box; dashed line: mean; and Q3: upper edge,) and whiskers correspond to “non-outlier” minimum and maximum values. Each panel shows results for respective molecules, namely (**A**) sTNF-R1; (**B**) sTNF-R2. The differences between groups with *p* < 0.05 are considered statistically significant.

The assessment of serum levels of both soluble TNF receptors revealed that in all groups, their mean levels were above the normal range (>0.5 ng/mL for sTNF-R1 and >2.5 ng/mL for sTNF-R2) [19]. Notably, in the PIMS group, the mean values were highest and significantly different from those in the COVID-19 group. However, although higher than the INFLAM group, they did not reach statistical significance in a comparison of PIMS vs. INFLAM. Also, the mean levels of sTNF-R1 and -R2 did not differ between the COVID-19 and INFLAM groups (Table 3, Figure 3).

The associations between the individual levels of selected immunomodulators were assessed in all patients. Very strong correlation (r = 0.9, at *p* < 0.01) was observed between serum concentrations of both soluble TNF receptors, as well as between IL-6 and either sTNF-R1 or sTNF-R2 (Table 4).

Furthermore, the individual serum levels of ADA, IL-6, sTNF-R1, and -R2 in all patients were also tested in regard to their possible association with molecular markers of cardiovascular injury—troponin I, NT-proBNP, and D-dimer. It was found that serum levels of troponin I revealed a statistically significant correlation with concentrations of both sTNF-R1 and sTNF-R2, whereas NT-proBNP was found to correlate only with levels of sTNF-R2. No other significant associations were observed either in the case of D-dimer, ADA, or IL-6 (Table 5).

## 3. Discussion

Our study has shown for the first time the significant increase in soluble receptors for TNF in patients with PIMS and that this increase significantly correlates with cardiovascular complications in these patients. However, it is unclear why these complications appear after several weeks ofCOVID-19, especially since the latter has usually a mild or even asymptomatic course. One can speculate that this is the consequence of an immature immune system in children, where the cytokine storm, typical for severe COVID-19 in adults, is relatively mild and develops rather as the prolonged inflammatory response. This assumption could be supported by the observations of increased levels of pro-inflammatory cytokines, mainly IL-6, but also TNF [6,20], which although lower when compared to those in adults’ COVID-19, are still elevated above the normal levels. Together with the outstanding vascular tropism of coronavirus via angiotensin-converting enzyme (ACE)-2 and, possibly, dipeptydyl peptidase (DPP)-4 [21,22], they may promote chronic inflammation in the vascular system and, finally, lead to the exacerbation and development of severe cardiovascular complications. One cannot exclude that the increased levels of ADA, which at least in our study were similar in the COVID-19 and PIMS groups, could also be involved in that process.

The most intriguing finding is the increased secretion of soluble receptors for TNF. The first possible explanation is based on canonical cellular response to the stimulus. The cell exposure to TNF may lead to increased expression of membrane receptors for this cytokine, which in turn is followed by the increased shedding of their soluble forms [23]. The latter are considered as the natural modulators of the response to TNF.

Also, it is plausible that the extensive damage of cardiomyocytes contributes to increased levels of soluble TNF receptors in circulation. Presumably, this scenario may result from either the direct action of various cardiotoxic agents or indirect mechanisms, including coronavirus-mediated endothelial injury of coronary arteries with subsequent myocardial ischemia [22]. Therefore, it may even be independent from high levels of TNF. However, further studies are needed to confirm the aforementioned hypotheses.

It is noteworthy that the exact role of TNF and its receptors in various cardiovascular complications remains unclear. Surprisingly, several studies have shown that in some patients with systemic inflammatory conditions, the therapeutic inhibition of TNF by a monoclonal antibody (e.g., infliximab) led to the severe worsening of heart function [23,24]. The observed phenomenon may be explained by the dual action of the TNF molecule that results from the substantially different function of its both receptors.

Accordingly, TNF-R1 is constitutively expressed on the surface of most cells. It belongs to the family of death receptors and its triggering by TNF leads to the formation of a death-inducing signaling complex, the activation of the caspase pathway, and the induction of programmed cell death. However, in the presence of some specific adaptor proteins, including TNF receptor associated factor 2 (TRAF2), the inhibitor of apoptosis (IAP), and receptor-interacting protein (RIP), the alternative pathways involving c-Jun N-terminal kinase (JNK) and nuclear factor (NF)-κB could be activated, leading to cell survival and the production of pro-inflammatory cytokines [23].

In contrast to the aforementioned, the expression of TNF-R2 is restricted mainly to immune cells but also endothelial cells and cardiomyocytes, and it is induced upon stimulation with pro-inflammatory factors. Binding of TNF to TNF-R2 activates the canonical and non-canonical signaling pathways with NF-κB and phosphatidylinositol-3-kinase (PI3K), which promote cell proliferation and survival [23]. Furthermore, TNF-R2, also in its soluble form, was identified as the molecule involved in a unique mechanism called reverse signaling. In this mechanism, TNF-R2 binds to the membrane-bound TNF and indirectly induces mitogen-activated protein kinase (MAPK) pathways, thus resulting in an immunosuppressive effect on TNF-expressing inflammatory cells, mainly T lymphocytes and monocytes [23,25].

Such complexity of TNF signaling may suggest that the molecular targeting of the TNF-dependent pathways could offer some therapeutic potential for patients with PIMS. Notably, the TNF-focused strategies may selectively block the pathogenic mechanisms only [23]; however, future research should clarify the safety and specificity of such approaches.

There are several limitations of our study. The first and main limitation is its proof-of-concept nature with the single time point of analysis, which certainly will require further research. The next issue is the very small number of patients involved. This was due to the low occurrence of symptomatic COVID-19 in children and outstanding rarity of PIMS (0.1% of children with coronavirus) [2]. Obviously, the small study groups significantly reduced the observation sensitivity and allowed for the detection of only strong effects or differences. Hence, although no significant differences were observed in some comparisons, the small sample size may limit the ability to detect such subtle variations.

Also, the collected data did not reveal a normal distribution, thus increasing the risk of result distortion and unintended bias in their interpretation. Nevertheless, even despite aforementioned restrictions, we still were able to observe statistically significant differences between study groups in some parameters.

Another weak point of this report is the size and composition of the INFLAM group. It was very small and highly heterogeneous, actually involving patients sharing two common features, namely (1) the presence of inflammatory disease, either infectious or non-infectious, and (2) excluded COVID-19 etiology of inflammation. Hence, the INFLAM group was intended to be a kind of non-COVID-19 control that could serve as a comparator to the COVID-19 and PIMS groups. Nevertheless, one has to keep in mind that with the small size and high heterogeneity, the real scientific value of that group is rather anecdotal and should be considered as a form of screening to indicate some directions for future research. Accordingly, when considering our findings concerning soluble TNF receptors and heart injury in PIMS, the most interesting selection for further more detailed comparative studies may be the group of patients with some cardiovascular diseases, e.g., Kawasaki disease.

## 4. Materials and Methods

Thisstudy involved three small groups of children, (1) one with active coronavirus disease (COVID-19), (2) with post-COVID-19 pediatric inflammatory multisystem syndrome (PIMS), and (3) with various non-COVID-19 inflammatory (INFLAM) diseases. All study participants were recruited among children referred to the Department of Pediatrics, Allergology and Pulmonology at the University Hospital of the Medical University of Warsaw between December 2020 and December 2021 during the second-to-fourth wave of theCOVID-19 pandemic.

All procedures involving patients were performed according to the Declaration of Helsinki. Whenever possible, due to patients’ age, children and/or all their parents or legal guardians gave written informed consent to participate in the study, which was approved by the Ethics Committee at the Medical University of Warsaw (approval no. KB/212/2020, dated 14 December 2020).

The patients were qualified for the COVID-19 group based on the suspected or already diagnosed infection with coronavirus (SARS-CoV-2, variants alpha or delta). The presence of typical symptoms and the COVID-19 diagnosis were verified by polymerase chain reaction and specific IgM/IgG antibody testing.

The children recruited to the PIMS group conformed to specified inclusion criteria as follows: COVID-19 or asymptomatic infection (confirmed by specific antibody testing) within the last 6 weeks, continuous fever above 38.5 °C for at least 3 days, and the occurrence of clinical symptoms involving at least two systems, namely gastrointestinal tract (usually with abdominal pain, diarrhea, nausea and/or vomiting), respiratory system (with cough, chest pain, and/or dyspnea), cardiovascular system (chest pain, arrhythmia, hypotension, faint, edema), or skin (erythema, rash, itching) issues.

The non-COVID-19 inflammatory group involved children with various diseases of either infectious (bacterial or viral, except those caused by coronavirus) or non-infectious origin. The main inclusion criteria concerned typical symptoms of inflammation, e.g., increased body temperature, pain, edema, and some other disease-specific symptoms, as well as elevated laboratory markers of inflammation.

All children were subjected to standard clinical examination. Apart from a routine laboratory assessment, including blood morphology, basic biochemistry, and coagulology, additional blood tests were focused on the measurement of serum levels of inflammation markerC-reactive protein, D-dimer, and two molecular indicators of heart injury (troponin I and N-terminal prohormone of brain natriuretic peptide–NT-proBNP). Furthermore, 2 mL of serum sample was kept frozen at −80 °C until being used for immunological tests.

The concentrations of IL-6, ADA, and both soluble TNF receptors—sTNF-R1 and -R2—in serum samples were measured in duplicate, using respective enzyme-linked immunosorbent assay (ELISA) tests according to detailed protocols provided by the manufacturer. Human ADA ELISA Kit was purchased from Invitrogen/Life Technologies Co. (Carlsbad, CA, USA). Human IL-6 High Sensitivity ELISA was from Invitrogen/Bender MedSystems GmbH (Vienna, Austria), whereas both Human sTNF-RI and sTNF-RII ELISA Kits were purchased from Invitrogen/Life Technologies Co. (Frederic, MD, USA).

The statistical analysis was performed using DATA-tab software (2024) (https://datatab.net; DATAtab e.U., Graz, Austria) and MedCalc Software 18.11v (https://medcalc.org; MedCalc Software Ltd., Ostend, Belgium), for both URL accessed between October–November 2024.

The Shapiro–Wilk test was used to assess the distribution of analyzed variables. The basic demographic and clinical features of patients within study groups, namely the age, body temperature, tissue oxygenation, and selected laboratory parameters, were analyzed using descriptive statistics involving the calculation of the arithmetic mean, median, standard deviations, confidence intervals, and quartiles. Then, they were compared between groups using the non-parametric Kruskal–Wallis test. The occurrence of main symptoms, due to the small sample size, was compared between groups using Fisher’s exact test. The observed differences were considered statistically significant at *p* < 0.05.

The correlations between selected parameters, such as heart injury markers (D-dimer, troponin I, and NT-proBNP) and immunoregulatory molecules (ADA, IL-6, and sTNF-R1 and -R2), as well as cross-talk between the latter, were analyzed using Pearson’s correlation test with a significance level *p* < 0.05.

## 5. Conclusions

Our proof-of-concept observation may be a step forward to better understand the phenomenon of mild COVID-19 in children and the pathomechanism of its severe cardiovascular complications in PIMS. We hypothesize that, possibly, the delayed immune response to coronavirus infection may result in the activation of the TNF pathway in the heart, the overexpression of TNF receptors, and the shedding of their soluble forms (sTNF-R1 and -R2) into circulation. Alternatively, inflammatory reactions or cardiotoxic effects of the coronavirus itself may result in excessive damage of cardiomyocytes and the release of soluble TNF receptors. However, both scenarios require validation in future research.

As shown in previous studies, the increased levels of sTNF-R1 and -R2 are indicators of poor prognostics; nevertheless, their exact role and the mechanism of that increase remain unclear. Our findings suggest that the TNF pathway may be explored as a potential therapeutic target to prevent PIMS complications in children and, presumably, in severe COVID-19 in adults. However, due to high complexity of TNF-related signaling, the molecular targeting should be highly selective and block the pathogenic mechanism only. Therefore, this issue still requires further studies.

## Figures and Tables

**Table 1:** Characteristics of patient groups.

Parameter/Feature	COVID-19 (*n* = 28)	PIMS (*n* = 14)	INFLAM (*n* = 18)
Demographics			
Sex (female/male; *n*=) Age (mean/median; ±SD)	14/14 5.6/1.5; ±6.2	6/8 7.8/8.5; ±4.8	10/8 6.1/5.5; ±4.8
Age groups (*n*=) 0–2 years 3–7 years 8–18 years	16 2 10	2 4 8	6 5 7
**Clinical features**			
Body temperature [°C] (mean/median; ±SD)	38.7/38.6; ±0.9	39.5/39.6; ±0.9	38.2/38.3; ±0.8
Tissue oxygenation (SpO_2_) [%] (mean/median; ±SD)	93.6/96.5; ±6.5 *^,#^	97.0/98.0; ±3.3 *	97.8/98.0; ±3.0 ^#^
Pulmonary injury (*n*=) Cardiac injury (*n*=) Kidney injury (*n*=)	7 1 * 0	3 11 *^,#^ 0	3 1 ^#^ 0
Other symptoms/comments	gastric symptoms—5 tracheobronchitis—1 otitis—1 urin. tract infection—1	mediastinitis—1 gastric symptoms—14 skin rash—9 conjunctivitis—7	resp. tract infection—8 gastric symptoms—4 vasculitis—2 otitis—2 urin. tract infection—2 arthritis—1

*^,#^: differences between respective groups are statistically significant.

**Table 2:** The mean/median values (±SD; 95% CI) of selected laboratory data in study groups.

Parameter [Normal Range]	COVID-19 (*n* = 28)	PIMS (*n* = 14)	INFLAM (*n* = 18)
WBC [4.5–13 × 10^3^/µL] PLT [150–400 × 10^3^/µL] CRP [0–10 mg/L] Hb [11–14 g/dL]	9.2/9.2; ±4.2; 7.7–10.8 289.9/291.5; ±82.1; 259.5–320.3	9.7/9.9; ±4.1; 7.5–11.8 232.4/233.5; ±124.1; 167.4–297.4	11.1/8.6; ±6.6; 7.9–14.2 277.2/236.0; ±111.3; 224.3–330.1
	2.4/1.6; ±2.2; 1.6–3.2 * 12.2/12.2; ±1.9; 11.5–12.9	15.9/15.5; ±10.2; 10.6–21.3 *^,#^ 11.4/11.4; ±1.3; 10.7–12.1	3.1/1.7; ±4.2; 1.0–5.1 ^#^ 11.8/12.4; ±2.1; 10.8–12.8
Creatinine [0.2–0.7 mg/dL] Troponin I [<19 µg/mL] NT-proBNP [<125 pg/mL] D-dimer [0–550 ng/mL]	0.4/0.3; ±0.2; 0.3–0.4 4.7/4.6; ±1.9; 2.8–6.6	0.6/0.4; ±0.4; 0.4–0.8 47.5/43.3; ±38.2; 26.0–69.1 *	0.3/0.3; ±0.1; 0.3–0.4 1.9/0.9; ±1.8; 0.3–3.5 *
	98/39; ±95; 15–181 * 907/730; ±566; 453–1360 *	8307/4571; ±9721; 2807–13807 *^,#^ 2572/2549; ±1777; 1606–3538 *	207/44; ±425; −132–549 ^#^ 1431/1108; ±1262; 557–2305

Abbreviations: SD—standard deviation; 95% CI—confidence interval; WBC—white blood cell; PLT—platelet; CRP—C-reactive protein; Hb—hemoglobin; NT-proBNP—N-terminal prohormone of brain natriuretic peptide; *^, #:^ differences between respective groups are statistically significant.

**Table 3:** The mean/median values (±SD; 95% CI) of selected immunological factors in study groups.

Parameter [Concentration]	COVID-19 (*n* = 28)	PIMS (*n* = 14)	INFLAM (*n* = 18)
ADA [pg/mL] IL-6 [pg/mL] TNF-R1 [ng/mL] TNF-R2 [ng/mL]	6.16/3.34; ±6.50; 3.76–8.56 9.78/3.68; ±12.26; 5.24–14.32 *	8.07/9.02; ±5.98; 4.94–11.20 20.12/14.65; ±16.63; 11.41–28.83 *	5.62/3.53; ±5.60; 3.05–8.19 14.43/6.64; ±14.04; 7.94–20.92
	4.51/4.09; ±1.64; 3.90–5.12 * 14.89/13.78; ±7.09; 12.26–17.52 *	8.37/7.69; ±5.47; 5.51–11.23 * 33.46/29.19; ±21.78; 22.05–44.87 *	5.84/4.75; ±4.01; 3.99–7.69 20.16/15.44; ±12.91; 14.20–26.12

Abbreviations: SD—standard deviation; 95% CI—confidence interval; ADA—adenosine deaminase; IL-6—interleukin-6; TNF-R—tumor necrosis factor receptor; *: differences between respective groups are statistically significant.

**Table 4:** The correlation between serum levels of selected immunoregulatory molecules.

Immunoregulator	ADA	IL-6	sTNF-R1	sTNF-R2
**ADA** **IL-6**	- r = 0.11; *p* = 0.56	r = 0.11; *p* = 0.56 -	r = −0.01; *p* = 0.97 r = 0.64; *p* < 0.01 *	r = 0.02; *p* = 0.92 r = 0.61; *p* < 0.01 *
**sTNF-R1** **sTNF-R2**	r = −0.01; *p* = 0.97 r = 0.02; *p* = 0.92	r = 0.64; *p* < 0.01 * r = 0.61; *p* < 0.01 *	- r = 0.90; *p* < 0.01 *	r = 0.90; *p* < 0.01 * -

*: correlation is statistically significant.

**Table 5:** The correlation between molecular markers of cardiovascular injury and selected immunoregulatory molecules.

Cardiovascular Marker vs. Immunoregulator	Troponin I	NT-proBNP	D-dimer
**ADA** **IL-6**	r = 0.11; *p* = 0.62 r = 0.16; *p* = 0.48	r = 0.34; *p* = 0.17 r = 0.20; *p* = 0.36	r = 0.33; *p* = 0.09 r = 0.27; *p* = 0.18
**sTNF-R1** **sTNF-R2**	r = 0.45; *p* = 0.04 * r = 0.49; *p* = 0.02 *	r = 0.29; *p* = 0.18 r = 0.40; *p* = 0.05 *	r = 0.22; *p* = 0.26 r = 0.17; *p* = 0.41

*: correlation is statistically significant.

## Data Availability

Due to law restrictions aimed to protect patient confidentiality, the original data are publicly not available; however, the data presented in this report are available from the corresponding author upon request.
